# Improving Video‐Based Prediction of Cerebellar Ataxia Severity Using a Pretrained Deep Learning Model

**DOI:** 10.1002/brb3.71781

**Published:** 2026-09-28

**Authors:** Katsuki Eguchi, Hiroaki Yaguchi, Hisashi Uwatoko, Yuki Iida, Moemi Yamada, Shinsuke Hamada, Kazunori Sato, Sanae Honma, Asako Takei, Atsushi Kawashima, Fumio Moriwaka, Toshiyuki Fukazawa, Ren Togo, Takahiro Ogawa, Miki Haseyama, Kenji Hirata, Kohsuke Kudo, Shinya Tanaka, Ichiro Yabe

**Affiliations:** ^1^ Department of Neurology Faculty of Medicine and Graduate School of Medicine Hokkaido University Sapporo Japan; ^2^ Division of AI Support for Medical Research Faculty of Medicine and Graduate School of Medicine Hokkaido University Sapporo Japan; ^3^ Hokuyukai Neurological Hospital Sapporo Japan; ^4^ Sapporo Neurology Hospital Sapporo Japan; ^5^ Faculty of Information Science and Technology Hokkaido University Sapporo Japan; ^6^ Department of Diagnostic Imaging Faculty of Medicine Hokkaido University Sapporo Japan; ^7^ Global Center for Biomedical Science and Engineering Faculty of Medicine Hokkaido University Sapporo Japan; ^8^ Medical AI Research and Development Center Hokkaido University Hospital Sapporo Japan; ^9^ Healthcare AIX Innovation Center (HAIXIC) Hokkaido University Sapporo Japan; ^10^ Department of Cancer Pathology Faculty of Medicine Hokkaido University Sapporo Japan; ^11^ Institute for Chemical Reaction Design and Discovery (WPI‐ICReDD) Hokkaido University Sapporo Japan; ^12^ Department of Surgical Pathology Hokkaido University Hospital Sapporo Japan

**Keywords:** cerebellar ataxia, deep learning, gait analysis, Scale for the Assessment and Rating of Ataxia (SARA)

## Abstract

**Background:**

Ataxia severity in degenerative cerebellar diseases (DCDs) is usually assessed with semi‐quantitative clinical rating scales such as the Scale for the Assessment and Rating of Ataxia (SARA), which are subject to variability. Deep learning‐based gait analysis offers an objective alternative, but labeled data in DCDs are limited. We investigated whether pretraining on gait videos from patients with Parkinson's disease (PD) could improve SARA score prediction in DCD.

**Methods:**

Gait videos from patients with DCD were processed using pose estimation, and the extracted time‐series keypoint coordinates were input to a transformer‐based model. Separate models were developed for the SARA total score and the posture and gait subscore (sum of Items 1–3). Models were pretrained on PD gait videos by supervised learning with the Movement Disorder Society–Sponsored Unified Parkinson's Disease Rating Scale (MDS–UPDRS) Part III axial subscore or by self‐supervised masked keypoint reconstruction. Performance was evaluated by leave‐one‐participant‐out cross‐validation (mean absolute error [MAE], coefficient of determination [*R* ^2^]), and between‐model differences by participant‐level Wilcoxon signed‐rank tests and bootstrap 95% confidence intervals (CIs).

**Results:**

We analyzed 75 patients with DCD and 141 with PD. For the SARA total score, MAE and *R* ^2^ were 1.91 ± 0.06 and 0.78 ± 0.01 with supervised pretraining, 1.95 ± 0.04 and 0.77 ± 0.01 with self‐supervised pretraining, and 2.04 ± 0.07 and 0.74 ± 0.02 with training from scratch; neither strategy significantly reduced participant‐level prediction error (*p* = 0.25 and 0.24). For the posture and gait subscore, only supervised pretraining significantly reduced prediction error (mean difference: −0.078; 95% CI: −0.127 to −0.031; *p* = 0.003).

**Conclusions:**

Pretraining on PD gait data modestly reduced SARA prediction error, reaching statistical significance for the posture and gait subscore with supervised pretraining. Pretraining across neurological disorders may help models acquire transferable representations of pathological gait, but its clinical significance requires validation in larger, multicenter datasets.

## Graphical abstract

A transformer‐based deep learning model predicted SARA scores from gait videos in patients with degenerative cerebellar diseases. Pretraining with Parkinson's disease gait data modestly reduced prediction error compared with training from scratch, reaching statistical significance only for the posture and gait subscore with supervised pretraining.

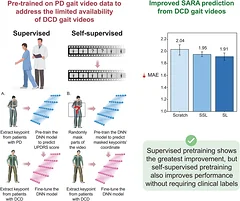

## 1 Introduction

Degenerative cerebellar diseases (DCDs) are a group of disorders characterized by progressive ataxia resulting from cerebellar and brainstem degeneration. Clinical rating scales, such as the Scale for the Assessment and Rating of Ataxia (SARA) (Schmitz‐Hübsch et al. 2006), are widely used to assess ataxia severity. However, these scales rely on semi‐quantitative judgments based on clinical observations and are therefore subject to inter‐rater variability and potential subjectivity (Subramony et al. 2005; Bürk et al. 2013). This limitation highlights the need for more objective and standardized measures of ataxia severity.

Advances in digital technologies have created new opportunities for the objective assessment of cerebellar ataxia. Pose estimation (Stenum et al. 2021), a computer vision technique that automatically identifies key body points, such as the head, trunk, and joints, from video recordings, has emerged as a promising tool (Martínez‐García‐Peña et al. 2025; Friedrich et al. 2025). This technique requires no specialized equipment or sensors attached to the patient and can be easily implemented at a low cost. Pose estimation has been increasingly applied to evaluate motor dysfunction in disorders such as Parkinson's disease (PD) (Shin et al. 2020; Park et al. 2021; Deng et al. 2024; Acevedo et al. 2025) and spinocerebellar ataxia (SCA) (L'Italien et al. 2024; Eguchi et al. 2024). Using keypoint coordinates extracted from pose estimation as input to deep learning models has been increasingly applied to analyze movement disorders and represents a promising approach (Kidziński et al. 2020; Brügge et al. 2023). We previously demonstrated that combining pose estimation with deep learning enables prediction of SARA scores from gait videos in patients with DCD (Eguchi et al. 2025). However, DCDs are relatively rare, and collecting a sufficiently large volume of labeled video data for training deep learning models is challenging. Because model performance generally depends on the size of the training dataset, the limited availability of labeled data poses a substantial barrier to achieving robust generalization and clinical applicability.

Recent advances in deep learning have demonstrated that large‐scale pretraining, including both supervised and self‐supervised learning, can substantially improve model performance when only limited task‐specific data are available. By leveraging datasets in related domains, such pretraining approaches enable models to learn transferable representations that can be effectively adapted to downstream tasks. These strategies have been successfully applied across multiple fields and provide a promising framework for improving performance in data‐limited clinical settings (Vanberlo et al. 2023, 2024). This approach may help address the challenge posed by the limited availability of training data in rare diseases by improving the prediction performance achievable with existing datasets, thereby supporting the development of more generalizable and reliable models for assessing ataxia severity.

## 2 Materials and Methods

### 2.1 Participants

This study included patients with DCDs and PD. The inclusion criteria were as follows:

1. Diagnosis of DCD or PD without evidence of secondary central nervous system disease.
2. Ability to walk without manual assistance or the use of a walker.

The exclusion criteria were as follows:

1. Severe visual or hearing impairment.
2. Cognitive impairment that limited the ability to follow instructions.
3. Symptomatic musculoskeletal disorders, such as acute fractures, spinal canal stenosis, or osteoarthritis.
4. History of drug or alcohol use associated with parkinsonism or ataxia.

The detailed diagnostic criteria for each disease category are provided in the Supporting Information. All procedures were conducted after obtaining written informed consent from the participants, and the study was approved by the Institutional Review Board of Hokkaido University Hospital (approval number: 022–0205).

### 2.2 Gait Video Recording

We videotaped the participants performing the timed up‐and‐go test (a 5 m round trip). All recordings were conducted in a flat hallway, and videos were captured from the frontal view. After the recording started, the participants were instructed to stand up from their chairs, walk toward the camera, turn around, walk back, and then sit back down on their chairs. The camera was mounted on a tripod placed 8 m from the chair and 3 m from the turning point. Videos were recorded using a consumer‐grade video camera (HDR‐CX470, Sony Corporation, Tokyo, Japan) at 30 frames per second (fps) with a resolution of 1280 × 720 pixels in the MP4 format. Participants were permitted to use a cane or handrail if needed. Video recordings of patients with PD were obtained in the on‐drug state. Three videos were recorded for each participant, using the same protocol. Participants were allowed to rest adequately between the recordings.

For some patients with DCD, multiple visits were scheduled to monitor longitudinal changes. During these follow‐up visits, gait videos were recorded using the same procedure, and the recordings were included in subsequent analyses to increase the size of the training dataset.

### 2.3 Clinical Assessment

The severity of ataxia in patients with DCD was assessed using SARA (Schmitz‐Hübsch et al. 2006). The severity of motor symptoms in patients with PD was evaluated using the Movement Disorder Society–Sponsored Revision of the Unified Parkinson's Disease Rating Scale (MDS–UPDRS) Part III (Goetz et al. 2008). All clinical assessments were performed at the time of the video recordings.

### 2.4 Keypoint Extraction Using a Pose Estimation Algorithm

We used the OpenPose library (Cao et al. 2021), a deep learning‐based pose estimation algorithm, to extract keypoints from the recorded gait videos. OpenPose provides two‐dimensional (2D) coordinates of 25 keypoints for each person in every video frame, yielding 50 values per frame (*X* and *Y* coordinates for each keypoint). The keypoints included the eyes, ears, shoulders, elbows, wrists, hips, knees, ankles, heels, big toes, and little toes (bilaterally), as well as the nose, neck, and mid‐hip.

The keypoint coordinate matrices obtained from each video were used as inputs for the deep learning model, as described in the following section.

### 2.5 Deep Learning Model

We aimed to predict SARA scores by inputting the extracted keypoint coordinates into a transformer‐based deep learning model. The transformer architecture, originally developed for natural language processing (Vaswani et al. 2017), uses self‐attention mechanisms to capture dependencies within sequential data. Transformer‐based models have recently demonstrated strong performance in various time‐series analyses (Zhou et al. 2021; Zerveas et al. 2021). In this study, we adopted a transformer‐based approach to model the temporal dynamics of gait‐related keypoint sequences. The detailed structure of the deep learning model is provided in the Supporting Information.

### 2.6 Model Pretraining Strategies

The primary aim of this study was to investigate whether pretraining using gait videos from patients with PD could improve the performance of a deep learning model in predicting SARA scores from a limited DCD dataset. We evaluated two pretraining strategies: Supervised pretraining using MDS–UPDRS Part III scores and self‐supervised pretraining without labels. The overall pretraining strategies are illustrated in Figure 1.

**FIGURE 1:**
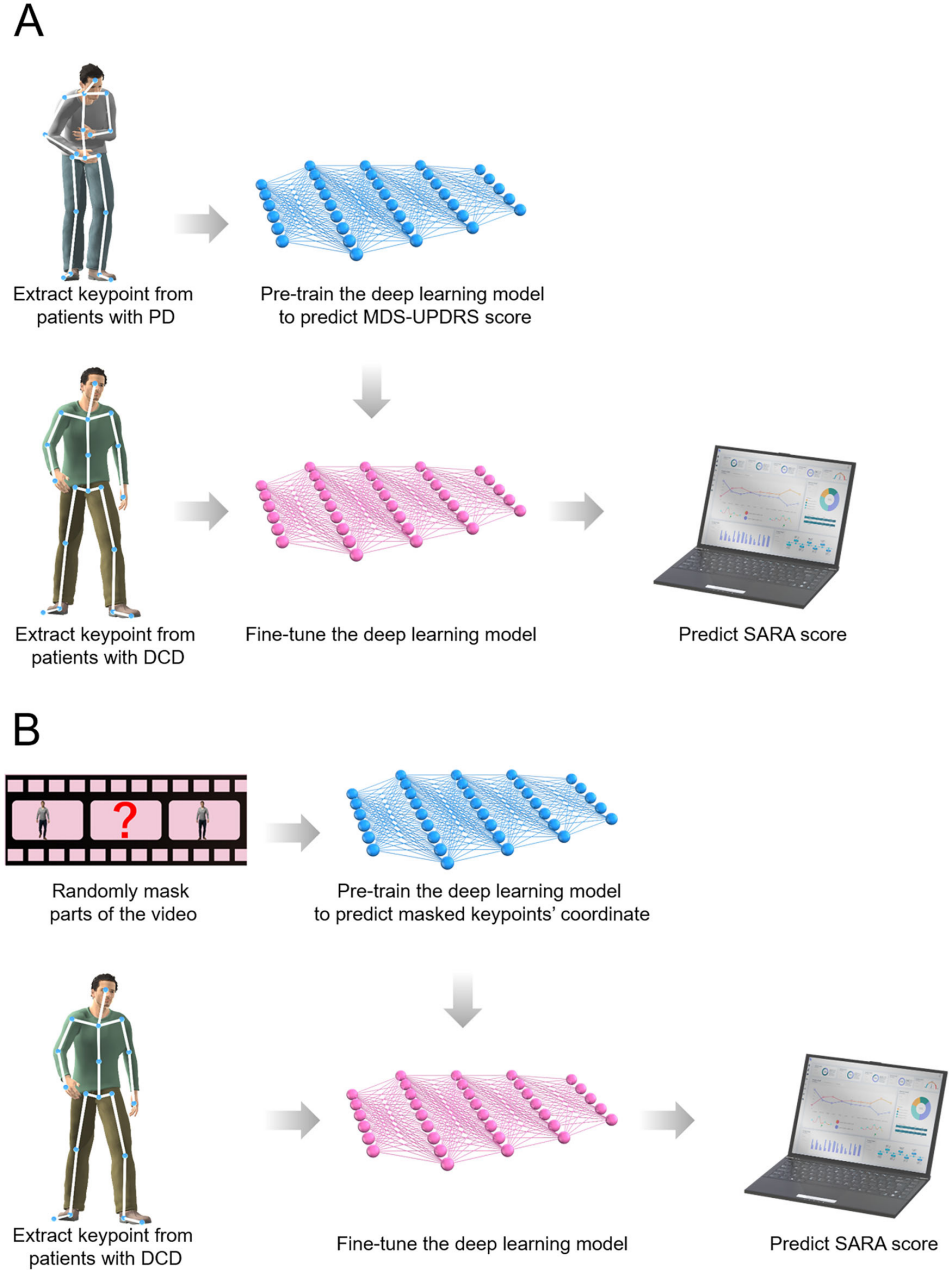
Overview of supervised and self‐supervised pretraining strategies. (A) Supervised pretraining strategy. Two‐dimensional keypoint coordinates were extracted from gait videos of patients with PD using OpenPose. The extracted keypoint sequences were input into a transformer‐based deep learning model. During pretraining, the model was trained to predict the MDS–UPDRS Part III axial subscore. After supervised pretraining, the pretrained model weights were fine‐tuned using gait data from patients with DCDs to predict the SARA score. (B) Self‐supervised pretraining strategy. Keypoint coordinates extracted from PD gait videos were masked over a temporally contiguous segment of frames, hiding all keypoints during the masked interval. The transformer‐based deep learning model was pretrained to reconstruct the missing keypoint coordinates from the surrounding visible frames without using clinical labels, thereby learning spatiotemporal gait representations in a self‐supervised manner. After pretraining, the model backbone was fine‐tuned using DCD gait data to predict the SARA score. DCD, degenerative cerebellar disease; MDS–UPDRS, Movement Disorder Society–Sponsored Revision of the Unified Parkinson's Disease Rating Scale; PD, Parkinson's disease; SARA, Scale for the Assessment and Rating of Ataxia.

In the scratch condition, the model parameters were randomly initialized and trained solely on the SARA prediction task using gait videos from patients with DCD. In the supervised pretraining condition, the same model architecture was first trained using gait videos from patients with PD to predict the MDS–UPDRS Part III axial subscore, defined as the sum of items 3.9–3.13 (Figure 1A). These items primarily reflect axial motor symptoms related to posture and gait. We selected this subscore as the supervisory signal because motor features such as tremor, rigidity, and speech impairment are difficult to infer reliably from gait videos. The pretrained weights were then fine‐tuned for SARA prediction in patients with DCD. In the self‐supervised pretraining condition, the model was trained using gait videos from patients with PD without providing any clinical labels. For each video sequence, a temporally contiguous segment of frames was masked, in which all keypoint coordinates were hidden. The model was then trained to reconstruct the full set of keypoint coordinates in the masked frames from the surrounding visible frames (Figure 1B).

After pretraining, all models were fine‐tuned on the SARA prediction task in DCD, and performance was compared across strategies. Additional details are provided in the Supporting Information.

### 2.7 Visualization of Learned Representations by Pretraining

To examine the effects of pretraining on the learned feature representations, we visualized the embeddings extracted from the transformer encoder backbone using principal component analysis (PCA). For each gait video of patients with DCD, the clip‐level features were aggregated into video‐level embeddings and projected onto a two‐dimensional space. The resulting representations were visualized as a scatter plot and colored according to the corresponding clinical severity scores. In addition, Spearman rank correlation coefficients were computed between each principal component and severity scores to quantitatively assess their associations. Detailed procedures are described in the Supporting Information.

### 2.8 Dataset Creation and Preprocessing

Because the SARA includes items assessing domains that are not directly observable from gait videos, such as speech disturbance and limb ataxia, we additionally developed models to predict the posture and gait subscore, defined as the sum of SARA items 1–3, in addition to the total SARA score.

Because the number of participants was relatively small, we employed a leave‐one‐participant‐out cross‐validation approach to evaluate the SARA prediction performance of the model. In each iteration, one participant was designated as the test patient. The keypoint coordinate data from the remaining participants were randomly divided into training (80%) and validation (20%) sets. The training set was used to optimize the model parameters, whereas the validation set was used to tune the hyperparameters, such as the number of training epochs. Using the parameters that showed the best performance on the validation set, the model generated a predicted SARA score for the keypoint data of the test patient. This procedure was repeated until each participant once served as the test patient. Importantly, there was no overlap between the participants used for model training and those used for testing to prevent data leakage. For participants with multiple study visits, all visits were assigned to the test set when the participant was selected as the test subject, and the predictions were evaluated separately for each visit.

The *X*‐ and *Y*‐coordinates of the extracted keypoints were standardized by subtracting the mean and dividing by the standard deviation computed from the training data in each cross‐validation fold. Keypoints that were not detected by OpenPose were treated as missing values. After normalization, the missing values were imputed with zeros.

### 2.9 Model Training and Fine‐Tuning for SARA Score Prediction

Because the shortest gait video was approximately 10 s, we used 10 s segments of keypoint data as inputs to the deep learning models. Each 10 s segment contained 300 frames (30 fps), and each frame contained 50 coordinate values; accordingly, the input to the model was a 300 × 50 matrix. During training, a 10‐s segments was randomly sampled from each gait video for each epoch. Different temporal segments were sampled across epochs to improve generalization. For each initialization condition, either pretrained weights obtained from the supervised or self‐supervised strategies or randomly initialized weights in the scratch condition, the model was fine‐tuned to predict the neurologist‐rated SARA score corresponding to each input segment. The prediction error was computed as the mean squared error between the predicted and true SARA scores, and the model parameters were optimized by minimizing the loss using the Adam optimizer. To reduce catastrophic forgetting during fine‐tuning, only the regression head was trained during the first 20 epochs for the supervised and self‐supervised pretrained models, whereas the backbone parameters were kept frozen. In contrast, in the scratch condition, all model parameters were trained from the beginning. All other hyperparameter settings were kept identical across the scratch, supervised, and self‐supervised conditions to ensure a fair comparison. Detailed training procedures and hyperparameter settings are provided in the Supporting Information.

### 2.10 Evaluation of the Fine‐Tuned Model's Prediction Performance

After the models were fine‐tuned, their prediction performance was evaluated using test patient data. The following evaluation procedure was applied separately to models predicting the SARA total score and the posture and gait subscore. The inference workflow is illustrated in Figure 2. First, the keypoint coordinate sequence from each test patient was segmented into non‐overlapping 10 s clips. The model generated a predicted target score for each clip. The average of all clip‐level predictions within a single gait video was defined as the video‐level prediction (Figure 2A).

**FIGURE 2:**
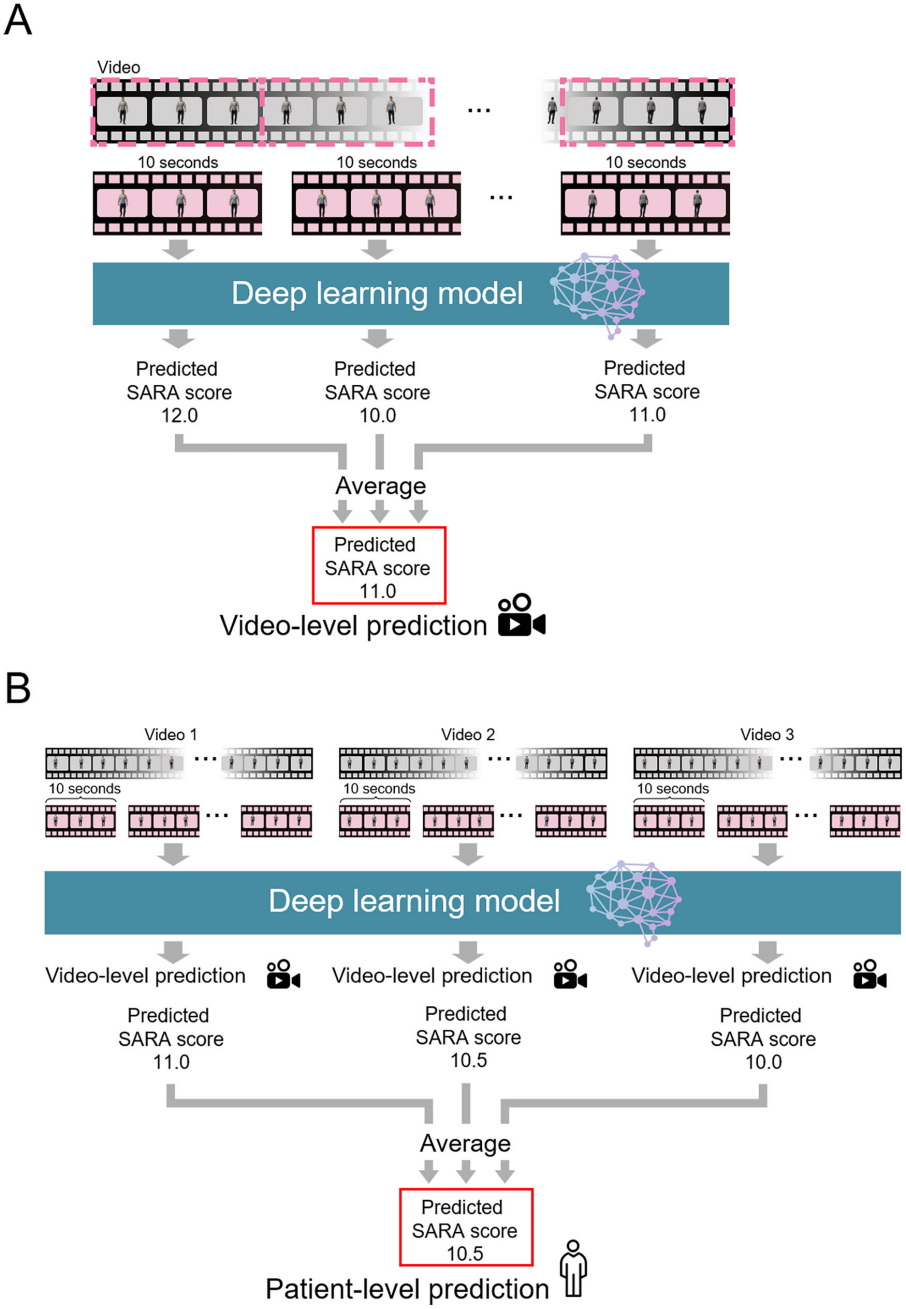
Inference workflow for video‐ and patient‐level SARA score prediction. (A) Video‐level inference. The keypoint coordinate sequence from each test gait video was segmented into non‐overlapping 10 s clips. The trained model generated a predicted SARA score for each clip. The average of all clip‐level predictions within a single gait video was defined as the video‐level prediction. (B) Patient‐level inference. Three gait videos were recorded for each participant, and a video‐level prediction was obtained for each video. The patient‐level predicted SARA score was calculated as the average of the three video‐level predictions and used for performance evaluation. SARA, Scale for the Assessment and Rating of Ataxia.

Three gait videos were recorded for each patient; therefore, three video‐level predictions were obtained for each participant. Patient‐level prediction was defined as the average of the three video‐level predictions (Figure 2B). The patient‐level predicted score was then compared with the corresponding neurologist‐rated score. For participants with multiple visits, patient‐level predictions and performance evaluations were performed separately for each visit.

To assess the agreement between the model‐predicted and neurologist‐rated scores, we computed the aggregated mean absolute error (MAE) and the coefficient of determination (*R* ^2^) using patient‐level predictions. These performance metrics were compared across the three pretraining strategies for both the SARA total score and the posture and gait subscore.

Because model performance may vary owing to the random initialization of model parameters and random assignment of training and validation datasets, the leave‐one‐participant‐out cross‐validation procedure was repeated 10 times for each training condition. The mean and standard deviation of MAE and *R* ^2^ across the 10 runs were calculated and reported separately for the SARA total score and the posture and gait subscore.

### 2.11 Statistical Analysis

To evaluate between‐model differences in prediction error, we performed participant‐level paired comparisons for both the SARA total score and the posture and gait subscore. For each participant, the absolute prediction errors were averaged across the 10 cross‐validation runs; for participants with multiple visits, absolute errors were averaged across visits so that each participant contributed a single value per condition. For each pretraining condition, participant‐level prediction errors were compared with those in the scratch condition using the Wilcoxon signed‐rank test and bootstrap estimation of the 95% confidence interval for the mean difference (10,000 resamples of participants). The same two procedures were applied uniformly to all comparisons for both prediction targets. Two‐sided *p* values < 0.05 were considered statistically significant.

## 3 Results

### 3.1 Participant Characteristics

We included 75 patients with DCD and 141 patients with PD in this study. Table 1 summarizes the demographic characteristics and clinical features of the participants, including their clinical rating scale scores. The breakdown of the diagnoses among patients with DCD was as follows: Multiple system atrophy, cerebellar variant (MSA‐C): 12; SCA6: 19; SCA3: 9; hereditary spinocerebellar degeneration without an identified pathologic gene: 5; SCA2: 5; SCA31: 5; SCA1: 3; SCA36: 2; dentatorubral‐pallidoluysian atrophy: 1; SCA42: 1; and sporadic adult‐onset ataxia: 13. Baseline SARA scores ranged from 1 to 20 (median: 13.5, interquartile range: 8.75–16.5), indicating that the cohort covered a spectrum from very mild to moderate ataxia among ambulatory patients. In the PD cohort used for pretraining, MDS–UPDRS Part III total scores ranged from 8 to 72 (median: 33.0, interquartile range: 24.0–41.0), and the axial subscale ranged from 0 to 15 (median: 4.0, interquartile range: 2.0–7.0).

**TABLE 1:** Demographics and characteristics of the participants.

	PD (*n* = 141)	DCD (*n* = 75)
Female, *n* (%)	64 (45.4)	37 (49.3)
Age, years	71.4 ± 8.2 (38–91)	63.8 ± 10.9 (33–84)
Disease duration, years	8.0 ± 5.4 (1.0–23.6)	9.4 ± 7.8 (1.0–40.1)
Hoehn and Yahr stage	2.9 ± 1.0 (1–4)	NA
MDS–UPDRS Part III total score	33.3 ± 13.0 (8–72)	NA
MDS–UPDRS Part III axial subscore	5.1 ± 3.4 (0–15)	NA
SARA	NA	12.5 ± 4.9 (1–20)
SARA posture and gait subscore	NA	5.9 ± 2.7 (0–10)

*Note*: Data are presented as *n* (%) or mean ± SD (range).

Abbreviations: DCD, degenerative cerebellar disease; MDS–UPDRS, Movement Disorder Society–Sponsored Revision of the Unified Parkinson's Disease Rating Scale; NA, not applicable; PD, Parkinson's disease; SARA, Scale for the Assessment and Rating of Ataxia.

Of the patients with DCD, 22 underwent two study visits, and 10 underwent three visits, resulting in repeated gait video recordings. At each visit, three gait videos were recorded, yielding 351 videos. All patients with PD underwent a single visit, with three videos recorded per participant, yielding 423 videos.

### 3.2 Visualization of Learned Representations

To examine how pretraining influenced the internal representations of the model, we visualized the feature embeddings extracted from the transformer encoder backbone prior to fine‐tuning using PCA. We first analyzed the embeddings derived from patients with PD using PCA (Figure 3A–C). In the supervised pretraining condition, the first principal component (PC1) showed a strong correlation with the MDS–UPDRS Part III axial subscore score (*ρ* = −0.893), which was expected given that the model had been explicitly trained using these clinical labels and indicated that the encoder effectively captured clinically relevant severity information. In contrast, the scratch model did not show an evident correlation (PC1: *ρ* = −0.049). Notably, the self‐supervised pretrained model also exhibited a substantial correlation with disease severity, although weaker than that of the supervised model, suggesting that clinically meaningful features could be learned even in the absence of explicit labels.

**FIGURE 3:**
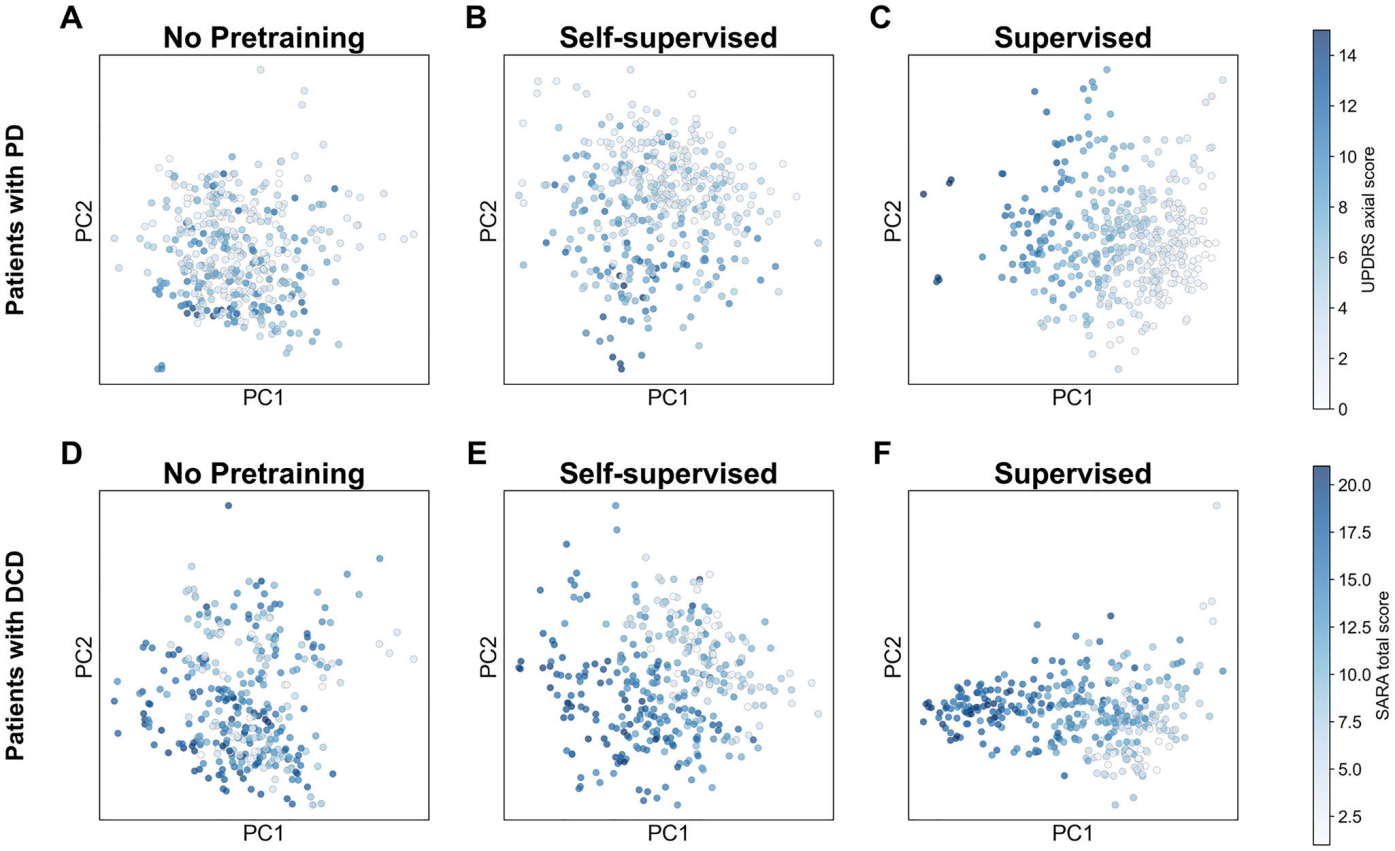
Principal component analysis of feature embeddings extracted from the transformer encoder before fine‐tuning. Two‐dimensional projections of video‐level embeddings are shown for different pretraining conditions and patient groups. The panels are arranged in a 2 × 3 layout: The top row represents patients with PD, and the bottom row represents patients with DCD. The left, middle, and right columns correspond to the scratch, supervised pretraining, and self‐supervised pretraining conditions, respectively (A–F). The data points are colored according to the corresponding clinical severity scores (MDS–UPDRS Part III axial subscore for PD and SARA for DCD). DCD, degenerative cerebellar disease; MDS–UPDRS, Movement Disorder Society–Sponsored Revision of the Unified Parkinson's Disease Rating Scale; PD, Parkinson's disease; SARA, Scale for the Assessment and Rating of Ataxia.

Next, we examined embeddings from the data of patients with DCD, which had not been used during pretraining (Figure 3D–F). In the scratch condition, no clear correlations were observed between the principal component axes and SARA scores (PC1: *ρ* = −0.203, PC2: *ρ* = −0.151), and no clear organization by severity was observed in the scatter plots. However, the self‐supervised pretrained model exhibited substantially stronger correlations (PC1: *ρ* = −0.638), with a visible gradient of severity scores along the principal component axes. The supervised pretrained model showed the strongest association (PC1: *ρ* = −0.775), with a clear separation between mild and severe cases along PC1. These findings indicate that both supervised and self‐supervised pretraining enabled the model to acquire representations that generalized beyond the pretraining domain and partially encoded disease severity in previously unseen patients with DCD.

### 3.3 Prediction Performance of Fine‐Tuned Models for SARA Scores

SARA prediction performance was evaluated using leave‐one‐participant‐out cross‐validation repeated 10 times. For each training condition, the mean and standard deviation of MAE and *R* ^2^ were calculated across runs.

Across 10 repeated cross‐validation runs, the supervised pretrained model achieved the lowest MAE (1.91 ± 0.06) and highest *R* ^2^ (0.78 ± 0.01), followed by the self‐supervised model (MAE: 1.95 ± 0.04, *R* ^2^: 0.77 ± 0.01), and the scratch model (MAE: 2.04 ± 0.07, *R* ^2^: 0.74 ± 0.02) in total SARA score prediction (Figure 4). In participant‐level paired comparisons, the reduction in absolute error relative to the scratch condition did not reach statistical significance for either strategy (supervised pretraining: mean difference −0.074, 95% CI [−0.205, 0.056], *p* = 0.25; self‐supervised pretraining: mean difference −0.057, 95% CI [−0.164, 0.048], *p* = 0.24). The performance of the model for SARA posture and gait subscore prediction was broadly comparable to that for the total score. Across the same 10 repeated cross‐validation runs, the supervised pretrained model achieved an MAE of 1.10 ± 0.02 and an *R* ^2^ of 0.73 ± 0.02, the self‐supervised model an MAE of 1.16 ± 0.02 and an *R* ^2^ of 0.72 ± 0.01, and the scratch model an MAE of 1.16 ± 0.02 and an *R* ^2^ 0.71 ± 0.02. In participant‐level paired comparisons, supervised pretraining reduced the prediction error of the subscore relative to training from scratch (mean difference −0.078, 95% CI [−0.127, −0.031], *p* = 0.003), whereas self‐supervised pretraining did not (mean difference −0.008, 95% CI [−0.071, 0.056], *p* = 0.95).

**FIGURE 4:**
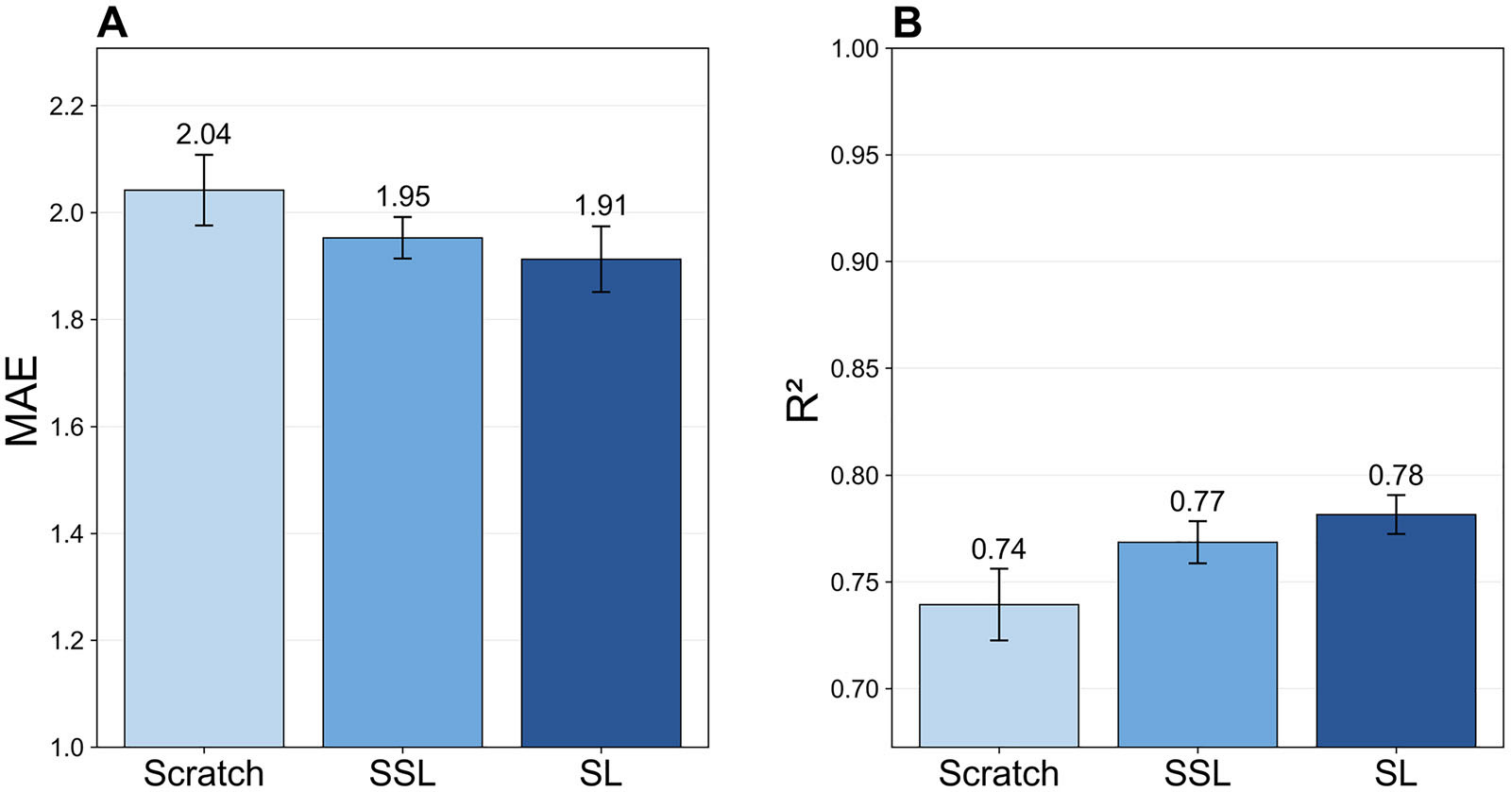
Prediction performance of fine‐tuned models for SARA score estimation. Bar plots show the mean absolute error (MAE) and coefficient of determination (*R* ^2^) across 10 repeated leave‐one‐participant‐out cross‐validation runs for the scratch, supervised pretraining, and self‐supervised pretraining conditions. Error bars represent standard deviations. Performance was evaluated using patient‐level predictions, and lower MAE and higher *R* ^2^ values indicate better predictive accuracy. MAE, mean absolute error; *R* ^2^, coefficient of determination; SARA, Scale for the Assessment and Rating of Ataxia; SL, supervised learning; SSL, self‐supervised learning.

## 4 Discussion

We investigated whether pretraining a deep learning model using gait videos from patients with PD could improve the prediction of ataxia severity in patients with DCDs. Both supervised pretraining using MDS–UPDRS Part III axial subscores as clinical labels and self‐supervised pretraining without labels showed the same direction of effect. We observed a statistically significant improvement in SARA posture and gait subscore prediction accuracy with supervised pretraining compared with training from scratch.

We used gait videos from patients with PD for model pretraining prior to fine‐tuning for SARA prediction in DCDs. We selected PD for two main reasons. First, although PD and DCD are distinct neurological disorders with different underlying pathophysiology, their gait patterns share several common features, including reduced gait speed, shortened stride length, and increased spatiotemporal variability (Shah et al. 2021; Mirelman et al. 2019; Buckley et al. 2018). These shared characteristics may allow the model to learn common representations of pathological gait during pretraining, facilitating improved prediction in DCD. Second, PD is considerably more prevalent than DCD, which enabled us to collect a larger pretraining dataset than would have been feasible with DCD alone. The choice of PD was therefore pragmatic rather than essential: Pathological gait data do not need to come from PD, and any sufficiently large gait dataset that captures relevant variation could in principle serve for pretraining. Indeed, pretraining on more diverse gait data—including healthy individuals as well as other neurological disorders—may allow the model to learn richer representations of human gait. In particular, because the present cohorts of both PD and DCD were weighted toward moderate disease severity, predicted scores for mildly affected patients tended to be overestimated. Incorporating gait data from healthy individuals into pretraining would populate the near‐normal end of the representation space and may therefore improve prediction accuracy in mildly affected patients whose gait is close to normal. More broadly, a large‐scale collection of gait video data from healthy individuals and patients with various neurological disorders alike may represent a valuable resource for developing objective severity assessment tools for rare ataxic disorders, in line with the concept of foundation models discussed below.

In this study, supervised pretraining using MDS–UPDRS Part III axial subscores resulted in better SARA prediction performance than self‐supervised pretraining. For the SARA total score prediction task, neither supervised nor self‐supervised pretraining resulted in a statistically significant reduction in prediction error compared with training from scratch. In contrast, for the SARA posture and gait subscore prediction task, supervised pretraining was the only approach that achieved a statistically significant reduction in prediction error compared with training from scratch. This performance difference may reflect the fact that supervised learning explicitly incorporates clinical labels that encode motor and gait‐related severity, thereby allowing the model to learn task‐relevant representations more directly during pretraining. This interpretation is further supported by PCA analyses, in which supervised pretraining produced a clearer severity gradient than self‐supervised learning. However, the collection of MDS–UPDRS scores requires time‐consuming clinical evaluations by trained specialists and may impose a substantial burden on clinicians, particularly when large‐scale datasets are required. In contrast, self‐supervised learning does not rely on disease labels or clinical rating scales. This label‐free property represents a major practical advantage in settings where standardized clinical assessments are difficult to obtain or not routinely collected. However, although the results of the PCA suggested that self‐supervised pretraining had captured representations related to the severity of gait disturbance, this did not translate into a statistically significant reduction in prediction error in the actual SARA prediction task. Moreover, even with supervised pretraining, the magnitude of the reduction in prediction error was small. These findings indicate that further validation in larger cohorts of patients with DCD is needed to determine whether pretraining can meaningfully improve SARA prediction performance. Accordingly, our findings should be interpreted as a proof of concept that gait representations learned from another neurological disorder are transferable to DCD—an interpretation independently supported by the PCA of the learned embeddings—rather than as evidence of a clinically relevant advantage of pretrained models. Larger multicenter cohorts will be needed to determine whether pretraining provides clinically meaningful gains.

Recent advances in deep learning have highlighted the potential of large‐scale pretraining and foundation models in various domains, particularly in natural language processing (Devlin et al. 2019), digital pathology (Xu et al. 2024), and clinical imaging (Yan et al. 2025). In the context of gait analysis, a large‐scale collection of gait video data may enable the development of more generalizable pretrained models. This study can be considered a preliminary step in this direction, as it demonstrates the transferability of gait representations across neurological disorders. However, developing truly generalizable models will require substantially larger and more diverse datasets and further systematic investigation.

Because the inclusion criteria required the ability to walk without manual assistance or a walker, the present cohort represented ambulatory patients with very mild to moderate ataxia (SARA 1–20), and patients who are unable to walk fall outside the scope of the present gait‐based approach. In such patients, severity assessment should instead rely on tasks that can be performed with the upper limbs, such as the nine‐hole peg test.

Several steps remain before the present approach can be applied in clinical practice. One of the major limitations is that this study was conducted using data from a single institution, and external validation using data from other institutions is essential to establish generalizability; a multicenter collaborative study with a standardized video recording protocol would be desirable for this purpose. In addition, the reliability of the model predictions, their minimal detectable change, and their sensitivity to longitudinal change should be evaluated. With improved prediction accuracy and established reliability, this approach could serve as a robust severity assessment method applicable not only to routine clinical evaluation but also to clinical trials.

This study has some other limitations. First, to ensure a fair comparison between the scratch, supervised pretraining, and self‐supervised pretraining conditions, identical model architectures and hyperparameter settings were used across all experiments. Therefore, the hyperparameters were not optimized separately for each training strategy. Strategy‐specific tuning can further improve the performance of all approaches; however, this requires additional systematic investigation. Second, the clinical spectrum of DCD included in this study was heterogeneous, and disease‐specific features that may influence gait were not explicitly modeled. For example, patients with SCA1 or SCA3 may exhibit spasticity in addition to ataxia, whereas those with MSA‐C may present with parkinsonian features that also affect gait. In the present study, gait severity was evaluated solely using the SARA score in patients with DCDs, without separately accounting for the potential contribution of coexisting motor features, such as spasticity or parkinsonism. The extent to which these additional motor features influence model predictions remains unclear and warrants further investigation in larger cohorts with more detailed phenotypic characterization. Future multicenter studies incorporating larger and more diverse cohorts, disease‐specific phenotyping, and strategy‐specific optimization are essential to address these limitations and further validate the proposed framework.

## Author Contributions

**Katsuki Eguchi**: conceptualization, methodology, software, formal analysis, funding acquisition, project administration, visualization, Writing – original draft, data curation, investigation. **Hiroaki Yaguchi**: conceptualization, writing – review and editing. **Hisashi Uwatoko**: conceptualization, writing – review and editing. **Yuki Iida**: resources. **Moemi Yamada**: resources. **Shinsuke Hamada**: resources. **Kazunori Sato**: resources. **Sanae Honma**: resources. **Asako Takei**: resources. Atsushi Kawashima: resources. **Fumio Moriwaka**: resources. **Toshiyuki Fukazawa**: resources. **Ren Togo**: methodology, writing – review and editing. **Takahiro Ogawa**: methodology, writing – review and editing. **Miki Haseyama**: writing – review and editing, supervision. **Kenji Hirata**: supervision, writing – review and editing. **Kohsuke Kudo**: supervision, writing – review and editing. **Shinya Tanaka**: supervision, writing – review and editing. **Ichiro Yabe**: conceptualization, validation, project administration, funding acquisition, writing – review and editing.

## Funding

This work was supported by the Japan Agency for Medical Research and Development (AMED) under the grant number JP256f0137006, the Japan Society for the Promotion of Science (JSPS) KAKENHI under the grant number JP25K19009, the Research Committee of Ataxia, Health Labour Sciences Research Grant, Ministry of Health, Labour and Welfare, Japan under grant number JPMH23FC1010, and Grants‐in Aid from the Research Committee of CNS Degenerative Diseases, Ministry of Health, Labour and Welfare, Japan under grant number JPMH23FC1008.

## Ethics Statement

The study was approved by the Institutional Review Board of Hokkaido University Hospital (approval number: 022–0205).

## Consent

All procedures were conducted after obtaining written informed consent from the participants.

## Conflicts of Interest

The authors declare no conflicts of interest.

## Supporting information




**Supporting File 1**: brb371781‐sup‐0001‐SuppMat.docx

## Data Availability

The raw gait video recordings analyzed in this study cannot be shared because they contain identifiable personal information of the participants. De‐identified derived data, including keypoint coordinate matrices and clinical rating scale scores, are available from the corresponding author upon reasonable request and subject to approval by the Institutional Review Board of Hokkaido University Hospital.
