# Supplementary material for: Improving Video‐Based Prediction of Cerebellar Ataxia Severity Using a Pretrained Deep Learning Model
Source: Brain Behav. 2026 Sep 28;16(10):e71781. doi: 10.1002/brb3.71781 (PMC13620301; doi:10.1002/brb3.71781)
Supplement: Supplementary file 1 [file BRB3-16-e71781-s001.docx]

**Improving Video-Based Prediction of Cerebellar Ataxia Severity Using a Pretrained Deep Learning Model**

Katsuki Eguchi^1,2,*^, Hiroaki Yaguchi^1^, Hisashi Uwatoko^1^, Yuki Iida^3^, Moemi Yamada^4^, Shinsuke Hamada^3^, Kazunori Sato^4^, Yoshiko^3^, Sanae Honma^3^, Asako Takei^3^, Atsushi Kawashima^4^, Fumio Moriwaka^3^, Toshiyuki Fukazawa^4^, Ren Togo^5^, Takahiro Ogawa^5^, Miki Haseyama^5^, Kenji Hirata^2, 6, 7, 8, 9^, Kohsuke Kudo^6, 7, 8, 9^, Shinya Tanaka^10, 11, 12^, Ichiro Yabe^1^

^1^Department of Neurology, Faculty of Medicine and Graduate School of Medicine, Hokkaido University

^2^Division of AI Support for Medical Research, Faculty of Medicine and Graduate School of Medicine, Hokkaido University

^3^Hokuyukai Neurological Hospital

^4^Sapporo Neurology Hospital

^5^Faculty of Information Science and Technology, Hokkaido University

^6^Department of Diagnostic Imaging, Faculty of Medicine, Hokkaido University

^7^Global Center for Biomedical Science and Engineering, Faculty of Medicine, Hokkaido University

^8^Medical AI Research and Development Center, Hokkaido University Hospital

^9^Healthcare AIX Innovation Center (HAIXIC), Hokkaido University

^10^Department of Cancer Pathology, Faculty of Medicine, Hokkaido University

^11^Institute for Chemical Reaction Design and Discovery (WPI-ICReDD), Hokkaido University

^12^Department of Surgical Pathology, Hokkaido University Hospital

**Supporting Information**

**Diagnostic criteria**

The diagnosis of Parkinson's disease (PD) was based on the United Kingdom Parkinson’s Disease Society Brain Bank criteria. [1]

Patients with multiple system atrophy (MSA), hereditary cerebellar ataxia (HCA), or sporadic adult-onset ataxia (SAOA) were included in the study. The diagnosis of MSA was based on consensus diagnostic criteria. [2] Only patients with the cerebellar variant (MSA-C) were included. HCA was defined as the presence of ataxic signs on neurological examination and a family history of similar ataxia syndromes in at least one first- or second-degree relative. The diagnostic criteria for SAOA were as follows: (1) presenting with progressive ataxia, (2) disease onset after 20 years of age, (3) no similar disorders in first- or second-degree relatives, (4) no established cause of ataxia (no ischemia, hemorrhage, or tumor of the posterior fossa; no alcohol abuse; no chronic intake of anticonvulsant drugs; no other toxic causes; no malignancies; no evidence of ataxia onset in association with encephalitis, sepsis, hyperthermia, or heat stroke; normal thyroid function), (5) no subacute ataxia onset, (6) and no probable MSA based on the consensus diagnostic criteria.^2^

**A detailed architecture of the deep learning model used in this study**

In this study, a Transformer-based deep learning model was used to predict the Scale for the Assessment and Rating of Ataxia (SARA) score from keypoint coordinates extracted from gait videos using OpenPose. The model used the Transformer encoder architecture as described in the original Transformer study. [3] This section describes the architecture of the Transformer-based deep learning model used in this study. An overview of the model architecture is shown in Figure S1.

The time-series data of the keypoint in 10-s clips (300 frames) were input into the DNN model. Each frame contained the X- and Y-coordinates of 25 keypoints, resulting in 50 values. Therefore, the input to the DNN model was a 300 × 50 matrix. First, each vector containing 50 coordinate values for each frame was input into a fully connected layer to obtain a new 128-dimensional vector. The following equation was used:

$\mathbf{u}_{t}=\mathbf{W}\mathbf{*x}_{t}+\mathbf{b}$ (1)

where **W** ∈ ℝ^128×50^ and **b** ∈ ℝ ^128^ are learnable parameters, **x**_t_ is a vector containing 50 coordinate values for an arbitrary frame (t = 1, …, 300), and **u**_t_ is the output of the fully connected layer in the corresponding frame and is a 128-dimensional vector. When the vectors for all 300 frames are input, **U** ∈ ℝ ^300×128^ = (**u**_1_, **u**_2_, …, **u**_300_) is the output of this layer.

Next, because the Transformer architecture is insensitive to the order of the input, a positional embedding is added to encode the sequential nature of the time series. These operations generate a new matrix **U’** ∈ ℝ^300×128^ as follows:

**U’ =** [**u**_1_, **u**_2_, …, **u**_300_] + **W**_pos_ (2)

where **W**_pos_ ∈ ℝ^300×128^ is positional embedding. Positional embedding was a learnable parameter that was initialized to zero. The resulting matrix was then input into the Transformer encoder.

The Transformer encoder comprises alternating layers of a multi-headed self-attention (MSA) block and a multilayer perceptron (MLP) block. Layer normalization (LN) was applied before MSA of the MLP block, and residual connections were applied after MSA of the MLP block. Self-attention is a technique used to capture the internal correlations between input features. Self-attention was calculated as follows: First, the input U’ was applied to the fully connected layers to generate a set of query matrix Q, key matrix K, and value matrix V. The weight coefficients were calculated using **Q** and **K**, and the generated weight coefficients were then used to calculate the weighted sum of the vectors in **V**. Self-attention was calculated as follows:

$Self Attention (\mathbf{Q, K, V})=Softmax\left( \frac{\mathbf{Q}\mathbf{K}^{T}}{\sqrt{d_{k}}} \right)\mathbf{V}$(3)

where d_k_ is the dimension of the feature vector of matrix **K** (i.e., d_k_ = 128 in this study), and Softmax is the softmax function, which rescales the elements of an input vector in the range of zero to one and sums to one.

MSA is an operation that generates h groups (called h-heads) of **Q**, **K**, and **V** matrices using the input matrix and calculates self-attention with each group of **Q**, **K**, and **V** matrices. Finally, the outputs of the h-heads were concatenated. The MSA is calculated as follows:

$\mathrm{head}_{i}=Self Attention (QW_{i}^{Q}, KW_{i}^{K}, VW_{i}^{V})$ (4)

$\mathrm{MSA}\left( Q, K, V \right)=concat (\mathrm{head}_{1}, \ldots, \mathrm{head}_{h})W_{o}$ (5)

where $W_{i}^{Q}$, $W_{i}^{K}$, $W_{i}^{V}$, and W_o_ are trainable parameter matrices and h is the number of MSA heads. In this study, h was set to four. Ultimately, combined with the LN and residual connections, the output of the MSA block y for input x is as follows:

$y=x+ MSA\left( \mathrm{LN}\left( x \right) \right)$ (6)

where LN denotes layer normalization.

The MLP block follows the MSA block. The MLP block consisted of two fully connected layers, and the activation function GELU was used in the first fully connected layer. The dimensionality of the input and output of the MLP block is 128, and the inner layer has a dimensionality of 512. Subsequently, the output of the MLP block was computed as:

$\mathbf{Z}=y+MLP(LN\left( y \right))$ (7),

where MLP denotes the operation of the MLP block, and the final output of the Transformer encoder is the same size as that of the input matrix (300 × 128). The same operation was repeated six times, with the output of the previous Transformer encoder as the input to the next Transformer encoder.

After repeating the operation of the Transformer encoder, the output matrix **Z** ∈ ℝ ^300×128^ was passed through a pooling-based regression head to generate the predicted score. The regression head consisted of a global adaptive concatenate pooling (GACP) layer, followed by a multilayer perceptron (MLP). The GACP layer computes the global adaptive average pooling and global adaptive max pooling across the temporal dimension, and concatenates the resulting vectors:

**z_avg** = AdaptiveAvgPool(Z) ∈ ℝ^128^ (8)

**z_max** = AdaptiveMaxPool(Z) ∈ ℝ^128^ (9)

**z_pool** = Concat(**z_avg**, **z_max**) ∈ ℝ^256^ (10)

where AdaptiveAvgPool and AdaptiveMaxPool compute the mean and maximum values across the temporal dimension, respectively. Global average pooling captures the overall movement pattern across all frames, whereas global max pooling captures the most salient features at any time point within the sequence. By combining both, we expect that the model captures complementary aspects of temporal dynamics.

The concatenated vector was then fed into a two-layer MLP to produce the final predicted score:

ŷ = **W**_2_ * ReLU(**W**_1_ * **z_pool** + **b**_1_) + **b**_2_ (11)

where **W**_1_ ∈ ℝ^32×256^, **b**_1_ ∈ ℝ^32^,**W**_2_ ∈ ℝ^1×32^, and **b**_2_ ∈ ℝ^1^ are learnable parameters. The output ŷ represents the predicted SARA or MDS-UPDRS Part III axial subscore.

**Preprocessing: Normalization and missing value handling**

OpenPose occasionally failed to detect keypoints that were recorded as zero values and treated as missing data. For standardization, the X- and Y-coordinates were normalized separately by subtracting the mean and dividing it by the standard deviation. Missing values (undetected keypoints) were excluded when calculating the mean and standard deviation. After normalization, the remaining missing values were imputed with zeros.

For supervised and self-supervised pretraining, normalization parameters were computed using all available PD training data. For downstream SARA prediction under cross-validation, the normalization parameters were computed exclusively from the training dataset in each fold and then applied to the corresponding validation and test datasets. This procedure prevented information leakage across the folds.

**Supervised Pretraining**

To leverage labeled clinical data from a related neurological disorder, we employed a supervised pretraining strategy in which the Transformer-based model was trained to predict motor severity scores from gait videos of patients with PD prior to fine-tuning on the SARA prediction task.

*Pretraining task*

In the supervised pretraining condition, the model was trained to predict the MDS-UPDRS Part III axial subscale score from keypoint coordinate sequences extracted from the gait videos of patients with PD. The model architecture used for supervised pretraining was identical to that used for the downstream SARA prediction task, including the Transformer encoder backbone with the GACP-based regression head described above.

*Pretraining dataset*

The pretraining dataset comprised gait videos recorded from 141 patients with PD using the same video recording protocol described in the Methods section of the main text. Each participant's gait video was processed using OpenPose to extract keypoint coordinates, yielding 50 values (X and Y-coordinates for 25 keypoints) per frame. A total of 423 gait videos were used for pretraining. Preprocessing for supervised pretraining was performed as described in the “Preprocessing: Normalization and Missing Value Handling” section. A10-second segment (300 frames at 30 fps) was randomly sampled from each gait video for each epoch, such that different temporal portions of the same video were used across epochs.

*Pretraining procedure*

The model was trained for 100 epochs with a mini-batch size of 64. The prediction error was computed as the mean squared error (MSE) between the model-predicted and neurologist-rated MDS-UPDRS Part III axial subscale scores. The Adam optimizer was used for parameter optimization, with a weight decay of 0.0001. The learning rate was set to 0.001 for epochs 1–50 and reduced to 0.0001 for epochs 51–100 using a step-wise learning rate schedule.

After pretraining, the model weights, including both the Transformer encoder backbone and the GACP-based regression head, were saved and subsequently used as the initialization for the SARA prediction task during fine-tuning.

**Self-supervised pretraining**

To leverage unlabeled gait data from patients with PD, we employed a self-supervised pretraining strategy based on the masked value prediction approach, inspired by the denoising autoregressive objective proposed by Zerveas et al. [4] We expect that this method will enable the model to learn meaningful representations from the temporal structure of multivariate pose keypoint sequences without requiring clinical labels.

*Pretraining task*

The self-supervised pretraining task consisted of reconstructing the masked portions of the input multivariate time series. Specifically, a binary mask **M** ∈ (0, 1)^w×m^ was applied to the input **X** ∈ ℝ^w×m^, where w = 300 represents the sequence length (10 seconds at 30 fps) and m = 50 represents the number of features (25 X-coordinates and 25 Y-coordinates of body keypoints). The masked positions in the input were replaced with zeros, and the model was trained to reconstruct the original values at the masked positions from the remaining visible context.

*Masking strategy*

We adopted a synchronized subsequence masking scheme in which all 50 keypoint coordinates were masked at the same time positions. The mask was generated using a stateful process based on geometric distribution, producing contiguous segments of masked and unmasked frames. Specifically, for each sample, the masked segments had a mean length of l_m frames, followed by unmasked segments with a mean length of l_u = ((1 − r) / r) × l_m frames, where r represents the overall masking proportion. This approach produces naturalistic patterns of temporal occlusion with varying segment lengths, unlike random Bernoulli masking, which produces isolated masked frames. We set r = 0.3 (30% of frames masked) and l_m = 90 frames (corresponding to approximately 3 s at 30 fps). These parameters were selected to ensure that the masked segments were sufficiently long to prevent trivial interpolation-based reconstruction while maintaining sufficient visible context for meaningful feature learning.

*Model architecture for Pretraining*

The Transformer encoder backbone used for self-supervised pretraining was identical to that described above for the SARA prediction task. During pretraining, the GACP-based regression head used for score prediction was replaced with a linear reconstruction layer. Specifically, a one-dimensional convolutional layer (Conv1d) with 128 input channels and 50 output channels (kernel size = 1) was applied to the Transformer encoder output at each time step to reconstruct the original 50-dimensional keypoint coordinate vector:

x̂_t_ = **W**_r_ * **z**_t_ + **b**_r_ (12)

where **W**_r_ ∈ ℝ^50×128^ and **b**_r_ ∈ ℝ^50^ are learnable parameters, **z**_t_ ∈ ℝ^128^ is the Transformer encoder output at time step t, and x̂_t_ ∈ ℝ^50^ is the reconstructed keypoint coordinate vector. This reconstruction layer operates independently at each time step and maps the Transformer encoder representations back to the original 50-dimensional input space.

*Loss function*

The reconstruction loss was computed only over the masked positions using the Huber loss (also known as the smooth L1 loss):

L = (1/|M|) Σ_{(t,i)∈M} H_δ(x̂_{t,i} − x_{t,i})

where M = ((t, i): m_{t,i) = 1} denotes the set of masked position indices, and H_δ is the Huber loss function defined as

H_δ(a) = { 0.5 a², if |a| ≤ δ

　　　　{ δ (|a| − 0.5 δ), if |a| > δ

with δ = 0.5. The Huber loss was chosen over the MSE because it is more robust to outliers; large reconstruction errors arising from noise or atypical keypoint positions are penalized linearly rather than quadratically, thereby reducing their disproportionate influence on the gradient updates.

*Pretraining procedure*

Pretraining was performed using the same normalization and missing value handling procedure described in the “Preprocessing: Normalization and Missing Value Handling” section. The model was trained for 100 epochs with a mini-batch size of 64 and a learning rate of 1 × 10−4. After pretraining, the full model weights were saved. For subsequent fine-tuning of the SARA prediction task, only the backbone weights were loaded, whereas the reconstruction head was discarded and replaced with the GACP-based regression head.

**Weight transfer strategy**

It is important to note that the weight transfer strategy differed between the supervised and self-supervised pretraining conditions. In the supervised pretraining condition, the model architecture, including both the backbone and GACP-based regression head, was identical to that used for the downstream task, allowing the transfer of all model weights (backbone + head). In the self-supervised pretraining condition, only the backbone weights were transferred because the pretraining head (Conv1d reconstruction layer) had a different architecture from that of the fine-tuning head (GACP + MLP regression head). Consequently, the regression head in the self-supervised condition was initialized randomly and trained from scratch during fine-tuning.

**Training the model for SARA prediction**

For each cross-validation fold, preprocessing (normalization and missing value handling) was performed as described in the “Preprocessing: Normalization and Missing Value Handling” section. The normalization parameters were computed using only the training dataset in each fold and then applied to the corresponding validation and test data to prevent data leakage.

We employed a leave-one-participant-out cross-validation method. First, one participant was selected as the test patient. Subsequently, the keypoint data obtained from the remaining participants were randomly split into training (80%) and validation (20%) sets. The training set was used to train the model, whereas the validation set was used to improve the hyperparameters, such as the number of training epochs. The models predicted the SARA scores of the test patients using the parameters that showed the best predictive performance in the validation data. This procedure was repeated so that all patients were tested once.

We trained the Transformer-based model described above using the following procedure. Because the shortest gait video was approximately 10 s long, 10 s of keypoint data were used as input for the DNN models. The 10-s video contained 300 frames, and the keypoint data contained 50 values for each frame; accordingly, the input into the model was a matrix measuring 300 × 50 in size. In the training of the models, 10 s of keypoint data were randomly cut from the entire keypoint data generated from each gait video to serve as the input for the model. For each epoch, 10 s of keypoint data were randomly cut from different ranges of the entire keypoint data. The model was trained to predict the SARA scores of the patients from whom the input keypoint data were generated. The model outputs the predicted SARA score for each input. The prediction error was calculated as the MSE loss between the model-predicted score and the neurologist-rated score. Subsequently, the model parameters were updated to reduce the MSE loss. To reduce catastrophic forgetting during fine-tuning, only the regression head was trained during the first 20 epochs for the supervised and self-supervised pretrained models, while the backbone parameters were kept frozen. From epoch 21 onward, all model parameters were jointly optimized. In contrast, in the scratch condition, all model parameters were trained from the beginning. The learning rates were scheduled as follows: 0.001 for epochs 1–20, 0.0001 for epochs 21–70, and 0.00005 for epochs 71–100. The total number of training epochs was set to 100. These hyperparameter settings were applied consistently across the scratch, supervised pretraining, and self-supervised pretraining conditions.

To increase the diversity of the training data and improve model generalization, four data augmentation techniques were applied: rotation, horizontal flipping, translation, and mixup.

*Rotation*

The target gait video was rotated at a predetermined angle and OpenPose was applied to the rotated video. The rotation angles were set to ± 1 °, ± 2 °, and ± 3 °.

*Horizontal flip*

A horizontal flip is an operation in which a video is generated with the left and right sides reversed. OpenPose was used for flipped videos.

*Translation*

Random offsets were added to the X- and Y-coordinates of the extracted keypoints to simulate positional shifts. For each training epoch, a random value was sampled from −50 to 50 for the X-axis and −25 to 25 for the Y-axis and applied uniformly to all frames within a clip.

*Mixup*

Mixup augmentation was applied by linearly combining pairs of randomly selected training samples and their corresponding labels. For two samples (x₁, y₁) and (x₂, y₂), mixed samples were generated as follows:

$Mixed coordinates= \lambda x_{1}+(1- \lambda)x_{2}$ (8)

$Mixed label= \lambda y_{1}+(1- \lambda)y_{2}$ (9)

where λ was sampled from a Beta distribution with parameters α = β = 10. Mixup was applied with a probability of 0.5 to each training batch.

All augmentation techniques were applied only to the training dataset and not to the validation or testing datasets.

Model training and related procedures were implemented in Python. We based our code on the open-source time series deep learning library Tsai (https://github.com/timeseriesAI/tsai) and adapted it for the specific needs of this study. The specific code used for this study, including custom modules and scripts, will be made available upon request.

**Visualization of learned representations**

*Embedding extraction*

For each model condition (scratch, self-supervised pretrained, and supervised pretrained), the Transformer encoder backbone was loaded with the corresponding weights. For the pretrained conditions, only the backbone weights were loaded from the saved pretrained models.

Each gait video from the patients with DSD was divided into non-overlapping segments of 300 frames (10 s at 30 fps). Segments shorter than 300 frames at the end of the video were discarded. Each segment was passed through the Transformer encoder backbone in evaluation mode, yielding an output tensor of shape (128 × 300). Global adaptive average pooling and global adaptive max pooling were applied across the temporal dimension to obtain two 128-dimensional vectors, which were concatenated to form a 256-dimensional clip-level embedding. Clip-level embeddings were averaged across all clips within a single video to obtain a single video-level embedding.

*Principal component analysis*

Principal component analysis (PCA) was applied to the matrix of video-level embeddings using scikit-learn (sklearn.decomposition.PCA) with n_components = 2. The first two principal components were computed, and the proportion of variance explained by each component was recorded. Spearman’s rank correlation coefficients were calculated between each principal component score and the corresponding clinical severity score.

**References**

1. Hughes AJ, Daniel SE, Kilford L, Lees AJ. Accuracy of clinical diagnosis of idiopathic Parkinson's disease: a clinico-pathological study of 100 cases. J Neurol Neurosurg Psychiatry 1992;55(3):181-184. doi:10.1136/jnnp.55.3.181

2. Gilman S, Wenning GK, Low PA, et al. Second consensus statement on the diagnosis of multiple system atrophy. Neurology 2008;71(9):670-676. doi:10.1212/01.wnl.0000324625.00404.15

3. Vaswani A, Shazeer N, Parmar N, et al. Attention is all you need. Adv Neural Inf Process Syst 2017;30:5998-6008.

4. Zerveas G, Jayaraman S, Patel D, Bhamidipaty A, Eickhoff C. A transformer-based framework for multivariate time series representation learning. In: Proceedings of the 27th ACM SIGKDD Conference on Knowledge Discovery & Data Mining; August 14-18, 2021; Singapore. ACM; 2021:2114-2124. doi:10.1145/3447548.3467401

**Figure S1**. Overview of the deep learning model

GACP, global adaptive concatenate pooling; SARA, Scale for the Assessment and Rating of Ataxia.
